# Drug Utilization Evaluation of Vancomycin among Patients in Jafar Ibn Auf Pediatric Hospital, 2018

**DOI:** 10.12688/f1000research.19370.2

**Published:** 2021-11-12

**Authors:** Tagwa A. M. Salih, Bashir A. Yousef, Mohamed A. M. Salih, Khalid S. Eltom

**Affiliations:** 1 Department of Pharmacology, Faculty of Pharmacy, Sudan International University, Khartoum, Khartoum, 11111, Sudan; 2 Department of Pharmacology, Faculty of Pharmacy, University of Khartoum, Khartoum, Khartoum, 11111, Sudan; 3 Department of Clinical chemistry, Faculty of Medical Laboratory, Karary University, Khartoum, Khartoum, 11111, Sudan; 4 Department of Pediatrics, Faculty of Medicine, National Ribat University, Khartoum, Khartoum, 11111, Sudan

**Keywords:** Vancomycin, Drug utilization evaluation, Pediatrics

## Abstract

**Background: **Vancomycin is an antibiotic of growing importance in the treatment of hospital-acquired infections; with a particular emphasis on its value in the fight against Methicillin-resistant *Staphylococcus aureus*. Increasing reports of Vancomycin resistance have raised concerns about the effectiveness of this drug. Drug utilization evaluation has an important role in controlling rational use of antibiotics to prevent the emergence of resistance.

**Methods:** We conducted a retrospective 6-months study at Jafar Ibn Auf pediatric hospital. Data including patient's demographics, diagnosis, Dosage regimen, and treatment duration were reviewed. The concordance of practice with the Hospital Infection Control Practices Advisory Committee (HICPAC) guidelines and principles of antibiotic therapy was assessed.

**Results: **127 medical records were reviewed in this study. Sepsis (29%) and Pneumonia (19.6%) were the most common indications. Culture test was requested in 20.5% of patients. Monitoring of serum creatinine was carried in 81.1% of patients. Based on HICPAC guidelines vancomycin was administered appropriately in 67.7% percent of cases. Considering the infusion rate, most of patients with specific order were received vancomycin in 1 hour.

**Conclusions: **The results showed that vancomycin was used empirically without subsequent adjustment of the antimicrobial agent according to culture and sensitivity data and lack of paying enough attention to the infusion rate and serum creatinine monitoring.

## Introduction

The majority of admitted inpatients are given antimicrobials as therapy or prophylaxis during their hospitalization. It has been shown that at least 50% of antimicrobial prescriptions are unnecessary. Antimicrobial over prescription increases the costs of health care, increases super-infection due to antimicrobial-resistant bacteria, and may increase the likelihood of an unwanted side-effects ^1^.

One critical challenge that health care faces is resistance to vancomycin, which is antibiotic that has a definite indications and a crucial role in treating infections in patients allergic to beta-lactam antibacterial medications or in bacterial infections that are resistant to other antibiotics ^2^. Vancomycin acts by inhibiting the earlier stage of development of the bacterial cell wall compared to beta-lactams, it blocks cell wall phospholipid synthesis by inhibiting transglycosylase enzymes ^3, 4^. Vancomycin has a narrow antibacterial spectrum against Methicillin-Resistant *Staphylococcus aureus* (MRSA), penicillin-resistant *Enterococcus* and *Streptococcus pneumonia* infections, thus is saved for these serious public health concerns ^5– 7^.

MRSA has become one of the predominant health care issues, and its resistance to vancomycin is growing. Cases of MRSA have increased in the US from 35.9% in 1992 to 64.4% in 2003, mainly due to inappropriate prescribing of broad spectrum antibiotics ^8^. Additionally, 10 to 30% of nosocomial infections in US were caused by vancomycin-resistant enterococci (VRE) ^9^. The increase in VRE has led to the development of recommendations for the use of vancomycin by the Hospital Infection Control Practices Advisory Committee (HICPAC) ^10^, a part of the Centers for Disease Control and Prevention (CDC), published in 1995. They indicate what constitutes appropriate and inappropriate usage of vancomycin, and advises physicians to use the guidelines to decrease the emergence of vancomycin-resistant strains ^11^.

Drug utilization evaluation (DUE) studies have an important role in controlling rational use of antibiotics to prevent resistance ^9^. Since we did not have any data regarding how rationally vancomycin is being prescribed in Jafar Ibn Auf Pediatric Hospital, we conducted this DUE study to determine the rate of rational use of vancomycin according to the standard guidelines.

## Methods

### Study design and setting

We performed a retrospective cross-sectional record-based study of vancomycin use in Jafar Ibn Auf Pediatric Hospital, Khartoum, Sudan. From January 2018 to June 2018

### Participants and study size

Medical records of all patients hospitalized during the study period were reviewed.

Inclusion criteria included: any pediatric patients receiving vancomycin during their hospitalization.

Exclusion criteria included: patients who received vancomycin orally because the infusion rate is one of the variables that should be measured and/or patients with incomplete data.

### Variables

Medical records were reviewed, and the following data was extracted using a check list (see extended data ^12^): dose of vancomycin; duration of infusion; duration of treatment with vancomycin; serum creatinine monitoring; culture and sensitivity test results; concurrent antimicrobials and nephrotoxic drugs; and adverse drug reactions.

### Data sources/ measurements

The study was carried out by reviewing all medical records of patients who were admitted and received vancomycin during the study period. A data collection form was used to gather patients’ information. Patients were grouped according to their age into: neonates (1–28 days), infants (1–24 month) and children (2–14 years). Appropriate or inappropriate use of vancomycin was classified according to the guidelines issued by HICPAC. The reasons for appropriate use include: the treatment of β-lactams-resistant gram-positive infections, treatment of patients allergic to β-lactams with gram-positive infections, discontinuation of vancomycin therapy when microbial cultures are negative, and empiric therapy in patients with risk factors; such as patients with co-morbidities and intensive care unit patient’s or confirmed gram-positive infections by culture. Cases in which the use was empiric in patients with risk factors has been justified by hospital epidemiology – due to a high prevalence of methicillin-resistant *Staphylococcus aureus* (MRSA).

### Statistical analysis

Statistical analysis SPSS version 20.0 was used to analyze the data. Continuous variables were analyzed using Student’s t-test. Chi-squire test was used to compare qualitative variables. P values less than 0.05 were considered to be statistically significant.

### Ethical statement

Ethical clearance (FPEC-07-2018) was obtained from the Ethical Committee of the Faculty of Pharmacy, University of Khartoum. Additional approval for checking the medical records was obtained from Jafar Ibn Auf Pediatric Hospital.

## Results

During the study period, 127 patients (61 females and 66 males) received vancomycin (Underlying data ^12^). As shown in Table 1, about a quarter of patients were hospitalized in a period of 11 to 15 days. Culture tests were performed in 20.5% of patients, with blood being the most common sample taken for microbiology study. Moreover, about 38.6% of patients received vancomycin for 4 to 7 days. ( Table 1)

**Table 1.** Demographic and clinical data of patients who received vancomycin.

Demographic & clinical characteristics	Number (Frequency %)
**Age group** 1–28 days(neonates) 1–24 month(infants) 2–14 years(children)	35 (27.56%) 42 (33.07%) 50 (39.37%)
**Hospital units** General Cardiology Respiratory Hematology Gastroenterology Nephrology Nursery	67 (52.76%) 7 (5.51%) 6 (4.72%) 3 (2.36%) 4 (3.15%) 5 (3.94%) 35 (27.56%)
**Outcome** Improved Failed Referred	119 (93.70%) 3 (2.36%) 5 (3.94%)
**Treatment duration** 1 to 3 days 4 to 7 days 8 to 14 days 15 to 21 days More than 21 days	13 (10.24%) 49 (38.58%) 46 (36.22%) 14 (11.02%) 5 (3.94%)

According to clinical indication, vancomycin was used to treat sepsis and pneumonia in 29% and 19.7% of patients respectively ( Table 2). However, around 55% of patients had received vancomycin without specific instructions from physicians to the nurses regarding the duration of infusion, while 26.8% and 18.1% of patients were receiving vancomycin with specific instructions stated from the physician in the medical records to give vancomycin for duration of 1 hour or 30 min, respectively ( Table 2).

**Table 2.** Indications for vancomycin and instructions by physicians regarding the duration of infusion use among the study participants.

Indications	Number (Frequency %)
Meningitis Pneumonia Sepsis Febrile neutropenia Osteomylitis Endocarditis Others	18 (14.17%) 25 (19.69%) 37 (29.13%) 2 (1.57%) 1 (0.79%) 1 (0.79%) 43 (33.86%)
Instructions regarding duration of infusion	Number (Frequency %)
Without any specific order Half an hour One hour	70 (55.12%) 23 (18.11%) 34 (26.77%)

Results for the monitoring of serum creatinine are presented in Table 3. Appropriate dose (dosing and frequency) was observed in 81% of the patients (including renal dose adjustment), the need for dose adjustment was essential in 21 patients, but only 11 cases were adjusted. Recorded adverse drug reactions and drug-drug interactions are shown in Table 3. Only 5% of patients developed nephrotoxicity, and furosemide was the most common prescribed drug recorded to have a potential drug-drug interaction.

**Table 3.** Monitoring of serum creatinine among the study participants, and adverse drug reactions and drug-drug interactions among the study participants.

Monitoring of serum creatinine	Number (Frequency %)
Once Twice weekly Not done	77 (60.63%) 26 (20.47%) 24 (18.90%)
Adverse drug reactions	Number (Frequency %)
Anaphylaxis Nephrotoxicity None	1 (0.79%) 6 (4.72%) 120 (94.49%)
Drug-drug interactions	Number (Frequency %)
Furosemide Amikacin NSAID Furosemide & Amikacin No	16 (12.60%) 3 (2.36%) 6 (4.72%) 2 (1.58%) 100 (78.74%)

NSAID – nonsteroidal anti-inflammatory drugs

According to the HICPAC guidelines, vancomycin use was considered appropriate in 86 participants (67.8%) ( Table 4). Of these, 27.6% of cases (35) were empiric therapy in patients with risk factors; such as patients with co-morbidities and intensive care unit patients. 22.8 % (29) was for the treatment of β-lactams-resistant gram-positive infections treatment, 12.6% (16) for confirmed gram positive infections by culture, 2.4% (3) for treatment of patients allergic to β-lactams with gram-positive infections and 2.4% (3) for discontinuation of vancomycin therapy when microbial cultures are negative. Moreover, there was a significant difference in the appropriateness of vancomycin use in the three groups based on age (neonates, infants and children), with P value= 0.015 ( Table 4).

**Table 4.** Number and percent of appropriate and inappropriate use of vancomycin based on age.

	Appropriate		Inappropriate	
Age	Number	Frequency %	Number	Frequency %
1 – 29 days 1–24 months 2–14years	30 28 28	85.7% 66% 56%	5 14 22	14.3% 34% 44%
**Total**	86	67.7%	41	32.3%

## Discussion

Vancomycin is an antibiotic used to treat serious infections in patients hypersensitive to β-lactam antibiotics, or those caused by bacteria resistant to β-lactams such as MRSA ^13^. In this study, sepsis (29%) and pneumonia (19.6%) were the most common indications for vancomycin use; a similar finding has been reported in Oman ^14^. On the other hand, an Iranian study showed that vancomycin was mostly used to treat febrile neutropenia (87.9%) and sepsis (74.5%) ^15^, which was similar to results reported in Hong Kong ^16 ^. Considering the duration of vancomycin therapy, the maximum duration (40 days) was prescribed in one patient suffering from pneumonia due to he suffered from other diseases; the minimum period of administration was one day. Most infections, including gram-positive bacteria, can be treated for less than 15 days with vancomycin, but the duration of treatment with vancomycin for endocarditis and osteomyelitis can last for up to an 8-week period ^17^.

Our study demonstrated that most patients received vancomycin empirically without following culture and sensitivity testing. The rate of requesting culture and sensitivity tests was low (20.5%), similar to Salehifar *et al.* In which culture and sensitivity test was performed in 30.1% of cases ^18^. The main reason for the low rate of requesting culture is that most patients had no health insurance to cover the cost of culture.

According to the HICPAC guidelines, 67.7% of the vancomycin prescriptions were appropriate; the reasons for its appropriate use included the treatment of β-lactams-resistant gram-positive infections, treatment of patients allergic to β-lactams with gram-positive infections, and discontinuation of vancomycin therapy when microbial cultures have been negative. Empiric therapy is also in patients with risk factors and confirmed gram-positive infections by culture. The rate of adherence to HICPAC recommendations was lower in our study compared to other trials, including Alfandari *et al.* ^19^ and Melo *et al.* ^20^ studies, in which the rate of appropriate use was 71% and 95% respectively. However, in Askarian *et al*’s study out of 200 vancomycin prescriptions, only 12 (6%) were considered appropriate ^21^; which was very low compared to our results.

Given the importance of infusion rate, and the occurrence of “red man syndrome” by vancomycin ^22^ (a hypersensitivity reaction characterized by flushing, erythema and pruritus, particularly of the upper body), the guidelines recommend that the time of infusion should be ≥ 30 minutes per 500 mg dose of vancomycin to avoid this infusion-related anaphylaxis–like reaction ^23, 24^. The results showed that in more than half of the patients (55%) there were no specific instructions regarding the duration of infusion. An anaphylactic reaction occurred in two patients.

Nephrotoxicity is one of the most common adverse drug reactions, so daily monitoring of serum creatinine and estimated creatinine clearance, in addition to ensuring the proper vancomycin dose can be effective while preventing renal toxicity ^25, 26^. Multiple risk factors influencing the occurrence of nephrotoxicity include; treatment duration beyond one week, pre-existing renal insufficiency, concurrent administration of nephrotoxic drugs, sepsis and critical illnesses, as well as infusion rate ^27, 28^. In this study, nephrotoxicity occurred in 4.7% of patients, although serum creatinine testing was performed in 81% of patients.

Concerning serum creatinine monitoring, guidelines recommend that it should be monitored at least twice weekly, and weekly for long term therapy ^29^. In this study, in 60.6% of patients; serum creatinine was monitored once (75% of them before initiating vancomycin therapy and 25% during treatment with vancomycin therapy), while only 20% of patients were monitored twice weekly. 19% of cases had no serum creatinine monitoring. In Oliveira and Ribeiro’s study, serum creatinine was monitored to 93.4% patients which was higher compared to our results ^30^.

Regarding dosing of vancomycin; in neonates, 15mg/kg is the suggested starting dose, then 10 mg/kg every 12 hours up to the first week after birth, followed by 10 mg/kg every 8 hours up to the age of one month ;for children >1 month 15 mg/kg every 8 hours (maximum daily dose 2 g) is recommended ^31^. Appropriate dosing was observed in 81% of the patients, which was higher than an Iranian study in which 52% of the participants were given an appropriate dose ^18^. Since 80% to 90% of vancomycin is excreted unchanged in the urine, dose adjustment is required in renal insufficient/failure patients ^24^. In this study, the need for dose adjustment was essential in 21 patients, but only 11 of cases were adjusted.

Regarding drug-drug interactions, the most common interactive drugs used were furosemide (12.6%), amikacin (2.4%) and non-steroidal anti-inflammatory drugs (NSAID) (4.7%). Two patients were receiving vancomycin, furosemide and amikacin concomitantly. All these medications have been reported to increase the risk of vancomycin-induced nephrotoxicity ^27^. Specially, the combination of aminoglycosides with vancomycin has shown to increase renal injury by 20% to 30% ^28, 29^.

Regarding the appropriate use of vancomycin, a significant variation in the appropriateness of vancomycin use was observed in the three groups based on age (Neonate, Infant, child), P value = 0.015. The most appropriate use was in neonates 85.7%. Dehghan *et al.* also showed a significant difference in the appropriateness of vancomycin use in three groups based on age (neonates, infants, and children) with P value of 0.017, however, the most appropriate use was observed in infants (68.1%) ^32^. The recorded irrational use of vancomycin in this study indicates deficiencies in utilization practices at the level of this hospital and perhaps the entire region. Such practices require further restrictive measures on vancomycin prescribing.

### Limitations of the study

One of the major limitations was the poor documentation in the medical records used. Thus, information such as therapeutic monitoring of serum vancomycin concentrations was not available; therefore, we exclude it from the variables to be measured and we recommend that it is essential to document serum concentration levels when using vancomycin.

## Conclusion

Our findings confirmed that more than 30% of patients received vancomycin without fulfilling the HICPAC criteria. Insufficient attention to monitor the rate of infusion of vancomycin and serum creatinine, in addition to low rate/lack of requesting culture and sensitivity testing are collectively indicated the reasons for inconsistence to HICPAC guidelines.

## Data availability

### Underlying data

Figshare: Drug utilization evaluation of vancomycin among patients in Jafar Ibn Auf Pediatric Hospital: a retrospective chart review, https://doi.org/10.6084/m9.figshare.8397350.v1 ^12^

- Vancomycin Data.xlsx (Extracted data from medical records)

### Extended data

Figshare: Drug utilization evaluation of vancomycin among patients in Jafar Ibn Auf Pediatric Hospital: a retrospective chart review, https://doi.org/10.6084/m9.figshare.8397350.v1 ^12^

- Check-list.doc (Checklist used for data extraction from medical records)

Data are available under the terms of the Creative Commons Zero “No rights reserved” data waiver (CC0 1.0 Public domain dedication)

## References

[1] Rehm SJ Sekeres JK Neuner E : Guidelines for Antimicrobial Usage. Pharm D Professional Communications Inc. Cleveland Clinic,2012–2013.

[2] Angaran DM : Quality assurance to quality improvement: measuring and monitoring pharmaceutical care. *AM J Hosp Pharm.* 1991;48(9):1901–1907. 10.1093/ajhp/48.9.1901 1928130

[3] Nightingale J Chaffe BW Colvin CL : Retrospective evaluation of vancomycin use in a university hospital. *Am J Hosp Pharm.* 1987;44(8):1807–1809. 10.1093/ajhp/44.8.1807 3631109

[4] Kaiser AB : Antimicrobial prophylaxis in surgery. *N Engl J Med.* 1986;315(18):1129–1138. 10.1056/NEJM198610303151805 3531863

[5] Mandell Douglas Bennett : Principles and Practice of Infectious Diseases. 8 ^th^ed. Philadelphia: Churchill Livingston;2015;377–400. Reference Source

[6] Svetitsky S Leibovici L Paul M : Comparative efficacy and safety of vancomycin versus teicoplanin: systematic review and meta-analysis. *Antimicrob Agents Chemother.* 2009;53(10):4069–4079. 10.1128/AAC.00341-09 19596875 PMC2764163

[7] Liu C Bayer A Cosgrove SE : Clinical practice guidelines by the infectious diseases society of america for the treatment of methicillin-resistant *Staphylococcus aureus* infections in adults and children. *Clin Infect Dis.* 2011;52(3):e18–55. 10.1093/cid/ciq146 21208910

[8] Klevens RM Edwards JR Tenover FC : Changes in the epidemiology of methicillin-resistant *Staphylococcus aureus* in intensive care units in US hospitals, 1992-2003. *Clin Infect Dis.* 2006;42(3):389–391. 10.1086/499367 16392087

[9] Center of Disease Control and Prevention guideline for Antibiotic Resistance Threats in the United States 2013. Reference Source

[10] Centers of Disease Control and Prevention for management of Multidrug-Resistant Organisms in Healthcare Settings 2007.

[11] Recommendations for preventing the spread of vancomycin resistance. Recommendations of the Hospital Infection Control Practices Advisory Committee (HICPAC). *MMWR Recomm Rep.* 1995;44(RR-12):1–13. 7565541

[12] Yousef B; Salih T Salih M : Drug utilization evaluation of vancomycin among patients in Jafar Ibn Auf Pediatric Hospital: a retrospective chart review. *figshare.* Dataset.2019. 10.6084/m9.figshare.8397350.v1 PMC8579476 34853680

[13] Tarar WL Farooq M Amin F : Drug Utilization Evaluation of Vancomycin in Teaching Hospitals of Lahore. *J Pharm Sci Res.* 2012;4(2):1728–33. Reference Source

[14] Al Za’abi M Shafiq S Al Riyami D : Utilization Pattern of Vancomycin in a University Teaching Hospital in Oman: Comparison with International Guidelines. *Trop J Pharm Res.* 2013;12(1):117–121. 10.4314/tjpr.v12i1.19

[15] Vazin A Japoni A Shahbazi S : Vancomycin utilization evaluation at hematology-oncology ward of a teaching hospital in iran. *Iran J Pharm Res.* 2012;11(1):163–170. 24250438 PMC3813098

[16] You JH Lyon DJ Lee BS : Vancomycin utilization at a teaching hospital in Hong Kong. *Am J Health Syst Pharm.* 2001;58(22):2167–9. 10.1093/ajhp/58.22.2167 11760919

[17] Ena J Dick RW Jones RN : The epidemiology of intravenous vancomycin usage in a university hospital. A 10-year study. *JAMA.* 1993;269(5):598–602. 10.1001/jama.1993.03500050076029 8240468

[18] Salehifar E Babamahmoodi F Alikhani A : Drug Utilization Evaluation of Vancomycin in a Referral Infectious Center in Mazandaran Province. *J Pharm Care.* 2014;2(2):55–59. Reference Source

[19] Alfandari S Levent T Descamps D : Evaluation of glycopeptide use in nine French hospitals. *Med Mal Infect.* 2010;40(4):232–7. 10.1016/j.medmal.2009.10.019 19959309

[20] Melo DO Sasaki M Grinbaum RS : Vancomycin use in a hospital with high prevalence of methicillin-resistant *Staphylococcus aureus*: comparison with Hospital Infection Control Practices Advisory Committe Guidelines (HICPAC). *Braz J Infect Dis.* 2007;11(1):53–6. 10.1590/S1413-86702007000100014 17625728

[21] Askarian M Assadian O Safaee G : Vancomycin use in a large teaching hospital in Shiraz, Islamic Republic of Iran, 2003. *East Mediterr Health J.* 2007;13(5):1195–201. 10.26719/2007.13.5.1195 18290414

[22] Polk RE Healy DP Schwartz LB : Vancomycin and the red-man syndrome: pharmacodynamics of histamine release. *J Infect Dis.* 1988;157(3):502–7. 10.1093/infdis/157.3.502 2449506

[23] Frymoyer A Hersh AL Benet LZ : Current recommended dosing of vancomycin for children with invasive methicillin-resistant *Staphylococcus aureus* infections is inadequate. *Pediatr Infect Dis J.* 2009;28(5):398–402. 10.1097/INF.0b013e3181906e40 19295465 PMC3101254

[24] Rossi S : Adelaide: Australian Medicines Handbook 2013. Reference Source

[25] Green K Schulman G Haas DW : Vancomycin prescribing practices in hospitalized chronic hemodialysis patients. *Am J Kidney Dis.* 2000;35(1):64–68. 10.1016/S0272-6386(00)70303-9 10620546

[26] Cappelletty D Jablonski A Jung R : Risk factors for acute kidney injury in adult patients receiving vancomycin. *Clin Drug Investig.* 2014;34(3):189–93. 10.1007/s40261-013-0163-0 24385282

[27] Carreno JJ Kenney RM Lomaestro B : Vancomycin-associated renal dysfunction: where are we now? *Pharmacotherapy.* 2014;34(12):1259–1268. 10.1002/phar.1488 25220436

[28] Rybak MJ Albrecht LM Boike SC : Nephrotoxicity of vancomycin, alone and with an aminoglycoside. *J Antimicrob Chemother.* 1990;25(4):679–687. 10.1093/jac/25.4.679 2351627

[29] Alberta Health Services: vancomycin Monitoring and Dosing Guidelines.2011.

[30] Melo DO Ribeiro E : Vancomycin use in a Brazilian teaching hospital: comparison with the Hospital Infection Controlpractices Advisory Committee Guidelines (HICPAC). *Braz J Infect Dis.* 2009;13(3):161–4. 10.1590/S1413-86702009000300001 20191189

[31] Broome L So TY : An evaluation of initial vancomycin dosing in infants, children, and adolescents. *Int J Pediatr.* 2011;2011:470364. 10.1155/2011/470364 22046192 PMC3199208

[32] Dehghan F Khorami N Taslimi Taleghani N : Drug Utilization Evaluation of Vancomycin in Pediatric Department. *Novel Biomed.* 2018;6(1):9–14. Reference Source

