# Supplementary material for: Drug Utilization Evaluation of Vancomycin among Patients in Jafar Ibn Auf Pediatric Hospital, 2018
Source: F1000Res. 2021 Nov 12;8:1708. Originally published 2019 Oct 1. [Version 2] doi: 10.12688/f1000research.19370.2 (PMC8579476; doi:10.12688/f1000research.19370.2)
Supplement: Peer review history

# Peer review: Reviewer response for version 2

**DOI:** 10.5256/f1000research.79346.r100128

**Published:** 2021-11-24

**Authors:** Ghulam Jilany Khan

**Affiliations:** 1 Faculty of Pharmacy, University of Central Punjab, Lahore, Pakistan; 2 State Key Laboratory of Bioelectronics, School of Biological Science and Medical Engineering (BME), Southeast University, Nanjing, China

The authors have addressed my comments. 

 I agree for its indexing.

Is the work clearly and accurately presented and does it cite the current literature?

Yes

If applicable, is the statistical analysis and its interpretation appropriate?

Yes

Are all the source data underlying the results available to ensure full reproducibility?

No

Is the study design appropriate and is the work technically sound?

Yes

Are the conclusions drawn adequately supported by the results?

Yes

Are sufficient details of methods and analysis provided to allow replication by others?

Yes

Reviewer Expertise:

Antimicrobials, Tumor Metastasis, Non Small Cell Lung Cancer, Diabetes, Basic and Clinical Pharmacology/Oncology, MDR Nanocareer Drug Deliver System

I confirm that I have read this submission and believe that I have an appropriate level of expertise to confirm that it is of an acceptable scientific standard.

# Peer review: Reviewer response for version 2

**DOI:** 10.5256/f1000research.79346.r100127

**Published:** 2021-11-12

**Authors:** Khalid O. Alfarouk

**Affiliations:** 1 Department of Evolutionary Pharmacology, and Tumor Metabolism, Hala Alfarouk Cancer Center, Khartoum, Sudan

Approved.

Is the work clearly and accurately presented and does it cite the current literature?

Yes

If applicable, is the statistical analysis and its interpretation appropriate?

Partly

Are all the source data underlying the results available to ensure full reproducibility?

Yes

Is the study design appropriate and is the work technically sound?

Yes

Are the conclusions drawn adequately supported by the results?

Yes

Are sufficient details of methods and analysis provided to allow replication by others?

Yes

Reviewer Expertise:

Pharmacology

I confirm that I have read this submission and believe that I have an appropriate level of expertise to confirm that it is of an acceptable scientific standard.

# Peer review: Reviewer response for version 1

**DOI:** 10.5256/f1000research.21233.r98730

**Published:** 2021-11-09

**Authors:** Khalid O. Alfarouk

**Affiliations:** 1 Department of Evolutionary Pharmacology, and Tumor Metabolism, Hala Alfarouk Cancer Center, Khartoum, Sudan

Dear Editor,

 Thank you very much for the current work titled: Drug Utilization Evaluation of Vancomycin among Patients in Jafar Ibn Auf Pediatric Hospital, 2018.

 The authors performed a drug utilization study to determine the rational use of vancomycin according to the standard guidelines at Jafar Ibn Auf Hospital. The topic is crucial and describes how vancomycin is prescribed, which provides the basis for utilizing an antimicrobial regimen against diseases. 

 However, before betting the house, some points should addressed in this manuscript:

- Was the study reported according to the STROBE statement for writing a cross-sectional study? 
- Regarding the exclusion criteria for the participants. Are there any other exclusion criteria in addition to who received vancomycin orally and/or patients with incomplete data?
- The authors should represent both the number and frequency % for each variable in the tables for the table.

Sincerely,

 Khalid

Is the work clearly and accurately presented and does it cite the current literature?

Yes

If applicable, is the statistical analysis and its interpretation appropriate?

Partly

Are all the source data underlying the results available to ensure full reproducibility?

Yes

Is the study design appropriate and is the work technically sound?

Yes

Are the conclusions drawn adequately supported by the results?

Yes

Are sufficient details of methods and analysis provided to allow replication by others?

Yes

Reviewer Expertise:

Pharmacology

I confirm that I have read this submission and believe that I have an appropriate level of expertise to confirm that it is of an acceptable scientific standard.

# Response

**Published:** 2021-11-09

**Authors:** Bashir Yousef

**Affiliations:** University of Khartoum, Sudan

**1- Was the study reported according to the STROBE statement for writing a cross-sectional study?**

**Reply: **Authors appreciate the reviewer for this valuable comment. We want to inform you that the study was conducted according to the STROBE statement.

**2- Regarding the exclusion criteria for the participants. Are there any other exclusion criteria in addition to who received vancomycin orally and/or patients with incomplete data?**

**Reply: **The authors would like to thanks the reviewer for this important question about the exclusion criteria for the participants. As we mentioned in this study, the only exclusion criteria were patients who received vancomycin orally and/or patients with incomplete data.

**3- The authors should represent both the number and frequency % for each variable in the tables for the table.**

**Reply:** We thanks the reviewer for this suggestion that further simplify the tables.

**As a response:** All Tables in the manuscript were modified by adding the number of the participants and frequency % for each response or answer.

# Peer review: Reviewer response for version 1

**DOI:** 10.5256/f1000research.21233.r59899

**Published:** 2020-04-15

**Authors:** Ghulam Jilany Khan

**Affiliations:** 1 Faculty of Pharmacy, University of Central Punjab, Lahore, Pakistan; 2 State Key Laboratory of Bioelectronics, School of Biological Science and Medical Engineering (BME), Southeast University, Nanjing, China

Vancomycin is an important antimicrobial drug particularly against MRSA however its growing development of resistance against the microbes has made it an important drug for further studies and a higher level of understanding for its utility and mode of usage.

 In present study, the authors conducted a retrospective study at Jafar Ibn Auf Hospital which is one of the good health care facility for infectious and other diseases. The results concluded that 86 patents (67.8%) were receiving the Vancomycin therapy appropriately against Sepsis (29%) and Pneumonia (19.6%) as most common  indication.

1. The authors have reported that the therapy was given to a pneumonia patient for 40 days and alternatively it was recommended for another patient for one day only, here I would suggest the authors to update the record for this duration of one day as well and whether it was discontinued for some medical reason OR due to treatment success OR due to something else. As per my understanding Vancomycin therapy should not be given for that much limited duration.
2. The authors have reported that in most of the cases vancomycin therapy was given as an imperial treatment, and culture sensitivity testing rate is very low, The authors should explain (in discussion) why they have this much low rate of  culture sensitivity testing?
3. I highly recommend the acceptance of this manuscript for publication as it will provide the basis for antimicrobial regimen design against certain diseases.

Is the work clearly and accurately presented and does it cite the current literature?

Yes

If applicable, is the statistical analysis and its interpretation appropriate?

Yes

Are all the source data underlying the results available to ensure full reproducibility?

No

Is the study design appropriate and is the work technically sound?

Yes

Are the conclusions drawn adequately supported by the results?

Yes

Are sufficient details of methods and analysis provided to allow replication by others?

Yes

Reviewer Expertise:

Antimicrobials, Tumor Metastasis, Non Small Cell Lung Cancer, Diabetes, Basic and Clinical Pharmacology/Oncology, MDR Nanocareer Drug Deliver System

I confirm that I have read this submission and believe that I have an appropriate level of expertise to confirm that it is of an acceptable scientific standard.

# Response

**Published:** 2021-11-09

**Authors:** Bashir Yousef

**Affiliations:** University of Khartoum, Sudan

**1- The authors have reported that the therapy was given to a pneumonia patient for 40 days and alternatively it was recommended for another patient for one day only, here I would suggest the authors to update the record for this duration of one day as well and whether it was discontinued for some medical reason OR due to treatment success OR due to something else. As per my understanding Vancomycin therapy should not be given for that much limited duration.**

**Reply: **The authors would like to thank the reviewer for this important comment. This patient was suffered from many diseases that made him stay for 40 days in the hospital.

**As a response: **We have added the explanation in the discussion section

- Considering the duration of vancomycin therapy, the maximum duration (40 days) was prescribed in one patient suffering from pneumonia due to he suffered from other diseases.

**2- The authors have reported that in most of the cases vancomycin therapy was given as an imperial treatment, and culture sensitivity testing rate is very low, The authors should explain (in discussion) why they have this much low rate of  culture sensitivity testing?**

**Reply:** The authors appreciate this important suggestion and we agree about the importance of explanations of the low rate of culture. The main reason for this is high cost of the culture, and many patients couldn’t afford to do it.

**As a response: **we added the explanation in the discussion part

- The main reason for the low rate of requesting culture is that most patients had no health insurance to cover the cost of culture. 

**3- I highly recommend the acceptance of this manuscript for publication as it will provide the basis for antimicrobial regimen design against certain diseases**

**Reply:** We appreciate your words about the importance of this study.
